# CHALLENGES TO THE 10 GOLDEN RULES FOR A SAFE MINIMALLY INVASIVE SURGERY (MIS) INGUINAL HERNIA REPAIR: CAN WE IMPROVE?

**DOI:** 10.1590/0102-672020210002e1597

**Published:** 2021-10-18

**Authors:** Christiano CLAUS, Leandro Totti CAVAZOLLA, Marcelo FURTADO, Flavio MALCHER, Edward FELIX

**Affiliations:** 1 Minimally Invasive Surgery Department, Jacques Perissat Institute, Positivo University, Curitiba, Brazil; 2 Surgery Department, Federal University of Rio Grande do Sul, Porto Alegre, Brazil; 3 Minimally Invasive Surgery Institute, Jundiaí, SP, Brazil; 4 Department of Surgery Albert Einstein College of Medicine, Bronx, USA; 5 Department of Surgery Marian Regional Medical Center, Santa Maria California, USA

**Keywords:** Inguinal hernia, Minimally invasive surgery, Laparoscopy, Robotic, critical view, Golden rules, Hérnia inguinal, Cirurgia minimamente invasiva, Laparoscopia, Visão crítica, Robótica, Regras de ouro

## Abstract

**Background:**

Since publication of our paper “Ten Golden Rules for a Safe MIS Inguinal Hernia Repair” we have received many questions. As the authors, we feel it is important to address these topics as a follow-up to our paper.

**Aim:**

To discuss in more details the main points of controversy, review the rules and update de recommendations.

**Method:**

The questions and discussions came mainly over five rules, numbered 3, 5, 6, 7, 10. We analyzed all the comments about recommendations and update some technical principles.

**Results:**

Rule 3 - Removing normal fat plugs from the obturator canal is unnecessary and therefore is not recommended; Rule 5 - transection of the uterine round ligament (1 cm proximal to the deep ring) facilitates adequate dissection. When performed in this way it does not appear to be associated with complications; Rule 6 - transection of huge sacs are safer than over-dissection of the cord structures. Whether dissecting completely the sac or abandon the distal part it results in less postoperative seromas is an ongoing debate; Rule 7 - any retroperitoneal structure traversing the internal ring is or play a role like a hernia. Failing to identify and remove the lipoma will ultimately result in the patient experiencing a recurrence; Rule 10 - in TAPP peritoneum should preferably be closed with suture than tackes.

**Conclusion:**

10 Golden Rules emphasize the most important surgical tips and technical steps that allow the safe performance of MIS repairs of inguinal hernias, regardless the technique.

## RESUMO

**Racional:**

Desde a publicação de nosso artigo “Dez Regras de Ouro para o Reparo Seguro de Hérnia Inguinal MIS”, recebemos muitos questionamentos. Como autores, sentimos que é importante abordar esses tópicos como seguimento do artigo

**Objetivo:**

Discutir com mais detalhes os principais pontos de controvérsia, revisar as regras e atualizar as recomendações.

**Método:**

As dúvidas e discussões surgiram principalmente sobre cinco regras, numeradas 3, 5, 6, 7, 10. Analisamos todos os comentários sobre as recomendações e atualizamos alguns dos princípios técnicos.

**Resultados:**

Regra 3 - remoção dos plugs de gordura normais do canal obturador é desnecessária e, portanto, não é recomendada; Regra 5 - transecção do ligamento redondo do útero (1 cm proximal ao anel profundo) facilita a dissecção adequada e quando realizado dessa forma, não parece estar associada com complicações; Regra 6 - transecção de grandes sacos herniários é mais segura do que a dissecção excessiva das estruturas do cordão espermático e, se dissecar completamente o saco ou abandonar a parte distal, resulta em menos seromas pós-operatórios ainda é motivo de debate; Regra 7 - qualquer estrutura retroperitoneal que atravessa o anel interno é ou desempenha o papel como uma hérnia e deixar de identificar e remover o lipoma acabará resultando em recorrência; Regra 10 - na TAPP o peritônio deve ser fechado preferencialmente com sutura do que com tacks.

**Conclusão:**

As 10 Regras de Ouro enfatizam as dicas cirúrgicas e etapas técnicas mais importantes que permitem a realização segura de reparos MIS de hérnias inguinais, independentemente da técnica.

## INTRODUCTION

Since the publication of our paper “Ten Golden Rules for a Safe MIS Inguinal Hernia Repair using an anatomical concept as a guide”^2^, it has been downloaded more than 15,000 times and we have received many questions about the study as well as praise and criticism. As the authors, we feel it is important to address these questions and criticisms as a follow-up to our paper. It is important to emphasize that in the original paper we did not intend to exhaust the subject or to discuss all aspects of minimally invasive inguinal hernia repair and its anatomical and technical variation because it is impossible in a single paper. Our main objective was to establish what we believe to be the essential steps that if followed by surgeons would result in a successful repair and minimizing the risk of complications. We hoped that describing the MIS technique in a didactic and systematic way might increase the safe adoption of this approach that has previously been promoted by the hernia guidelines^5^ ^,^ ^16^ but is only utilized in 10-20% of repairs worldwide.

The objective of this new approach is to discuss in more details the main points of controversy, review the rules and update de recommendations. 

## METHOD

Our article published a year ago has become a reference both for standardizing the technique and for teaching MIS repair of inguinal hernias. However, some doubts and controversies about the rules have also been raised.

As the authors, we feel it is important to address these questions and criticisms as a follow-up to our paper. The questions and discussions came mainly over five rules, numbered 3, 5, 6, 7, 10. Analyzing all the comments and understanding the possible improvements in the recommendations, we now update some technical principles in the previously five published rules. 

## RESULTS

### Rule 3:

#### Original writing

*Dissection should extend to at least the pubic symphysis and at least 2 cm below the pubis at Zone 2, in order to create sufficient space to accommodate an adequately sized mesh, that overlaps Direct and Femoral Triangles by at least 3-4 cm and will not be lifted by the distending bladder.*

#### Update

**FIGURE 1:**
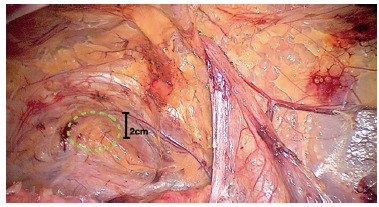
(Right groin) Dissection of Zone 2 extends to at least until 2 cm below the pubis (black marker). The green circle shows the area of the obturator foramen, which is usually filled with a normal fat plug. It does not seem advisable to extend the dissection further, unless an obturator hernia is suspected.

*There have been many questions and debates about how to handle zone 2 (the medial dissection), it’s limits and how to handle the obturator foramen. Although obturator hernias are extremely rare* ^*13*^ *we feel it is important to extend the dissection at least 2 to 3 cm below the pubis in order to achieve adequate coverage of the myopectineal orifice (MPO) so that the mesh will not be displaced with expansion of the bladder. By extending the dissection in this manner the surgeon will identify an obturator hernia if it exists and adequately cover it with mesh. As many have pointed out removing normal fat plugs from the obturator canal is unnecessary and may cause bleeding and therefore is not recommended (* *Figure 1* *).*

### Rule 5

#### Original writing

*Parietalization of the elements of the cord is considered sufficient when the peritoneum is dissected inferiorly until at least the level at which the vas deferens crosses the external iliac vein in Zone 3 and the iliopsoas muscle is identified posteriorly at Zone 1.*

*Comment: In women, round ligament of the uterus is usually closely adherent to the peritoneum. Transection of round ligament is then recommended, 1 cm proximal the deep ring in order avoid injury of genital branch of the genitofemoral nerve at this location.*

#### Update

**FIGURE 2:**
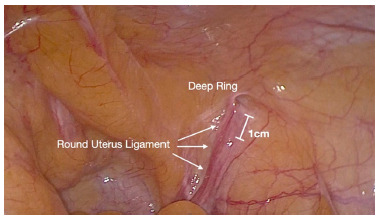
(Right groin) Peritoneum usually surrounds the round ligament of the uterus in the lower third. To facilitate dissection, the surgeon may choose to transect the ligament 1cm below the deep ring.

Many questions have been raised about transection of the round ligament when repairing inguinal hernias in the female patient. We feel it is important to transect the round uterus ligament in order to facilitate the parietalization of the peritoneum and achieve a proper dissection as well as for the correct positioning of the mesh. It is important that the transection takes place cephalic/proximal (at least 1cm bellow of internal ring) to the point where the genital branch of the genitofemoral nerve joins the round ligament and to ligate the vessels to prevent possible bleeding post-op (artery is normally obliterated but can persist). There is little evidence that transection at this point leads to any postoperative gynecological complications and it can be performed safely, if done in the matter we have suggested^14^ ^,^ ^15^ (Figure 2). If one wants to preserve the round ligament as done with the cord in males, the surgeon must be extremely careful to do an adequate parietalization in order to prevent displacement of the mesh when the peritoneal flap is closed in the TAPP repair or the CO_2_ is evacuated in the TEP repair. As well, an improper dissection can result in a poorly positioned mesh. 

### Rule 6

#### Original writing

*In large or inguinoescrotal hernias, it is recommended to transect and abandon the distal hernia sac within the scrotum.*

*An indirect hernia sac is usually dissected and reduced from the inguinal canal. When dealing with large hernia sacs or chronic and fibrotic ones, one may safely transect the hernia sac after safely identifying the elements of the spermatic cord. This decision is made to avoid excessive dissection of the cord elements thus avoiding injury to them. It is easier to deal with a pseudo-hydrocele postoperatively than with a hematoma of the scrotum, ischemic orchitis or injury to the spermatic cord.*

#### Update

**FIGURE 3:**
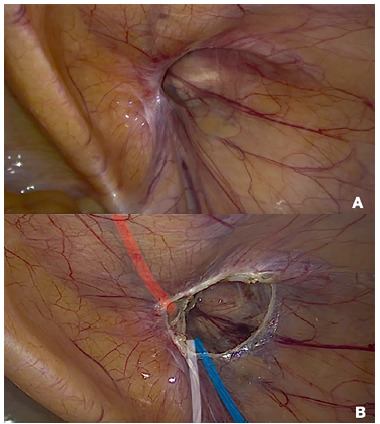
Right groin: A) Enlarged deep inguinal ring and large hernia with fibrotic hernia sac; B) circular incision of the peritoneum at the level of the deep ring performed as the first stage of the surgery (one of the technical possibilities). Distal part of the hernia sac is abandoned in the scrotum. Attention to the lower epigastric vessels, vas deferens and spermatic vessels (inverted Y represented in red, white and blue respectively)

In this rule we have discussed how to handle the indirect hernia sac. We have recommended that in cases where the indirect sac is extremely large, adherent and difficult to dissect completely from the cord structures that it should be partially and carefully separated from the cord, transected and the distal end be abandoned and left in the scrotum. Whether dissecting completely the sac or abandon the distal part it results in less postoperative seromas is an ongoing debate^7^ ^,^ ^12^. We feel however that for most cases transection of those huge sacs is safer than over-dissection of the cord structures and possible devascularization of the testicle and/or hematoma^6^ ^,^ ^9^. It is important to make the decision as early as possible in the procedure whether to dissect the sac completely or whether to transect it, so that unnecessary over the dissection of the testicular vessels and vas deferens can be avoided (Figure 3). It’s important to stress that for the vast majority of small and middle size indirect hernias the sac can be dissected without any complication, and the maneuver that we propose should be used only when the surgeon’s judgement is that there will be more harm to dissect a huge sac from the cord structures. 

### Rule 7

#### Original writing

*The deep inguinal canal should be explored during Zone 3 dissection in search of lipoma of the cord.*

*The so-called cord lipoma is an extension of retroperitoneal fat that usually runs laterally to the elements of the spermatic cord at the deep inguinal ring area. Often the simple visual inspection of the deep inguinal annulus does not clearly identify the presence of a lipoma. Any lipoma must be dissected and reduced from the inguinal canal. Untreated lipomas are a major cause of recurrence after laparoscopic repair.*

#### Update

We have emphasized the importance of ruling out a cord lipoma and how to handle it during MIS repair. Most surgeons have agreed but some argue that the lipoma is not a true hernia^10^. We feel that any retroperitoneal structure whether it be bladder or lipoma of the cord traversing the internal ring is or play a role like a hernia. The patient experiences the same symptoms and bulge. Failing to identify and remove the lipoma will ultimately result in the patient experiencing a recurrence. In the same way, it is very likely that the radiologist, in an eventual image exam, when detecting a fatty tissue passing through the deep inguinal orifice, will interpret it as a hernia recurrence. Those who argue against removal of the lipoma state that the dissection of lipomas may result in increased postoperative pain, especially in the testicle. There is little evidence to support this contention but there is ample evidence that leaving behind a lipoma may result in the patient requiring a second operative procedure^5^ ^,^ ^8^. 

### Rule 10

#### Original writing

*Deflation under direct visualization*

*In TAPP, suture closure of the peritoneum is recommended instead of tacking it closed because the latter technique may increase the potential for nerve injuries. Most surgeons agree that gaps, holes or tears of the peritoneum should be closed to reduce the risk of early bowel obstruction or mesh exposure.*

#### Update

The final controversy raised by our paper is how the peritoneum in TAPP should be approximated. Although there is one paper that suggests tacking the peritoneum is safe^1^ ^,^ ^11^, we feel that this technique is risky. Blind placement of penetrating fixation of the peritoneum, whether permanent or absorbable may unnecessarily injure the ilioinguinal and iliohypogastric nerves and therefore should be avoided. The more staples or tacks used in the fixation of the mesh or closure of the peritoneum, the greater the risk of postoperative pain^4^. We have suggested suture closure of the peritoneum, although technically more difficult to avoid this unnecessary risk.

## DISCUSSION

Despite the greater recent adoption of the MIS techniques for repair of inguinal hernias, still the majority of the surgeons have as first option the open surgery. In addition to lower direct costs, possibility of local anesthesia and safety of conventional surgery; the increased complexity of laparoscopic or MIS techniques, unusual anatomy and technically more demanding are the main reasons for this situation. Based on this, it seems fundamental to create a systematization of the operative steps, define the anatomical elements of reference as well as creating a universal language to the surgeons.

One of the major contributions to the evolution of laparoscopic cholecystectomy was the establishment and dissemination of the concept known as critical view of safety in order to reduce the incidence of biliary tract injuries. The same concept was recently introduced for inguinal repairs by Daes and Felix^3^. In the same direction, Furtado et al^4^ have published a new didactic proposal to understand (and teach) the anatomy of the posterior inguinal region and the definition of dissection zones, each one with its particularity.

In our previous paper (Ten Golden Rules for A Safe MIS Inguinal Hernia Repair Using a New Anatomical Concept as a Guide) we gathered these concepts and defined the most important surgical steps (do and don’t) for MIS repair of inguinal hernias, regardless of the technique (TAPP, TEP, ETEP, RTAPP). With well-defined steps presented, we aim to standardize MIS repairs and facilitate further safe dissemination of the technique. 

Since then, we have received many compliments but at the same time many doubts and questions about the proposed surgical steps. As the authors, we feel it is important to address these topics as a follow-up to our paper. The questions and discussions came mainly over five rules, numbered 3, 5, 6, 7, 10. Analyzing all the comments and understanding the possible improvements in the recommendations, we now update some technical principles in the previously five published rules. 

Although the desire and effort to establish a definitive systematization of the surgical technique some technical aspects of MIS inguinal repair still remain controversial as the best management of transversalis fascia; to close or not the defect (especially for direct hernias); type and shape of the mesh; etc. The 10 golden rules are a guide, a roadmap, a checklist for the surgeon wanting to perform MIS inguinal hernia repair. The order in which the steps are taken may be varied according to the patient’s hernia and surgeon’s preference but should not compromise the principles of the critical view of myopectineal orifice (MPO)^16^.

## CONCLUSION

We feel that the rules outlined are the basic steps required to perform a safe MIS inguinal hernia repair whether done TAPP, TEP, e-TEP, or r-TAPP, and that they may be adjusted in the future as we gain more knowledge. By facilitating the learning, understanding and applying this concept, we hope that more and more surgeons and consequently patients can be benefited.
